# Tagging polyketides/non-ribosomal peptides with a clickable functionality and applications

**DOI:** 10.3389/fchem.2015.00011

**Published:** 2015-02-20

**Authors:** Xuejun Zhu, Wenjun Zhang

**Affiliations:** ^1^Department of Chemical and Biomolecular Engineering, University of California, BerkeleyBerkeley, CA, USA; ^2^Energy Biosciences Institute, University of California, BerkeleyBerkeley, CA, USA; ^3^Physical Biosciences Division, Lawrence Berkeley National LaboratoryBerkeley, CA, USA

**Keywords:** natural product labeling, bioorthogonal chemistry, alkyne-azide cycloaddition, polyketides, non-ribosomal peptides, biosynthesis

## Abstract

Bioorthogonal chemistry has recently emerged to be one of the most powerful tools in drug discovery and chemical biology. The exploration of it has successfully advanced the field of natural product research. In this Perspective, we survey current strategies for the installation of chemical handles into the molecular scaffolds of several major classes of natural products, including polyketides (PKs), non-ribosomal peptides (NRPs), and their hybrids. By tagging these natural products with chemical handles and coupling them with subsequent bioorthogonal reactions, researchers have visualized and studied the mode of action of natural products, as well as synthesized derivatives with better pharmaceutical properties. We conclude this Perspective by considering two questions: is there a general way to synthesize tagged PKs/NRPs? Does natural product labeling have a broader impact in the field of natural product research beyond current known applications?

## Introduction

Nature's small molecules derived from microbes, plants, and animals have played an enormous role in the history of medicinal and pharmaceutical chemistry. In the past two decades, more than one third of small molecule-based drugs approved by the US Food and Drug Administration (FDA) were natural products or their derivatives (Newman and Cragg, 2012). Collectively, natural products have been widely used to treat nearly all human health conditions, including but not limited to infectious, neurological, cardiovascular, metabolic and oncological diseases (Butler, 2008). It has been estimated that most major classes of antibiotics and over 70% of anti-cancer small molecule treatments are natural products, their derivatives or mimics (Newman and Cragg, 2012). Whereas, combinatorial chemistry fails to deliver leads that form the basis for the development of successful new drugs, medicinally active natural products have functional group arrays and scaffold architectures that offer advanced platforms for the optimization of compound activity profiles.

Although the pharmaceutical value of natural products has been widely recognized, it is still challenging to transform medicinally active natural products into drugs (Clardy and Walsh, 2004). Firstly, sufficient quantities of natural products are required to fully characterize their chemical and biological properties. Considering the small amount of natural products that are typically obtained by biological fermentation, the preparation process can be time-consuming and labor-intensive. Secondly, most medicinally active natural products exert inhibitory functions on specific protein targets, and the identification of these protein targets is essential for successful drug development. Since the weak specificities and affinities between natural products and protein targets complicate the interpretation of experimental data, fishing for the protein targets of natural products from an entire proteome depends on the development of sensitive and reliable methods (Carlson, 2010). Thirdly, natural product-based new drug screening requires a library of natural products and their derivatives. However, current approaches to generate such library are limited, often relying on complicated chemical syntheses (Thirumurugan et al., 2013).

**Figure 1:**
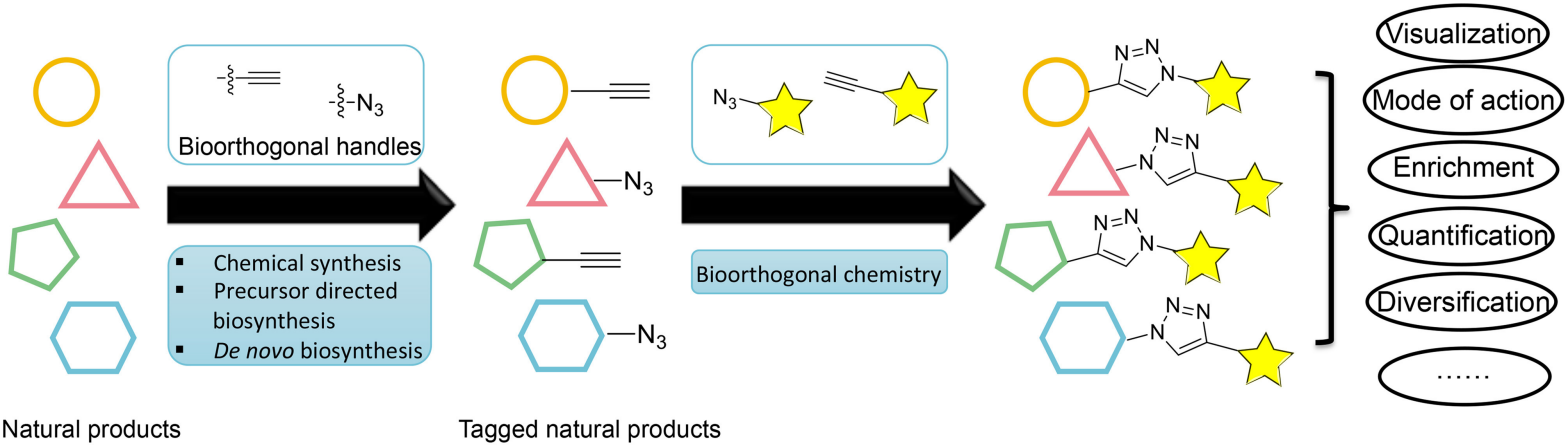
**Overview of different strategies for labeling natural products and their applications through bioorthogonal chemistry**.

An emerging enabling technology for expanding the natural product research toolkit is tagging natural products with a unique chemical handle that can be subjected to further bioorthogonal chemical transformations (Figure 1). The recent development of bioorthogonal chemistry is paving the way for new innovation in biology; it has produced new tools for labeling macromolecules, enabling the selective visualization and study of proteins, glycans, nucleic acids, and lipids (Prescher and Bertozzi, 2005; Grammel and Hang, 2013). Analogously, tagging natural products with a unique chemical handle will enable the visualization, enrichment, quantification, and mode of action study of natural products through bioorthogonal chemistry. This approach would have a profound impact on our ability to address challenging questions in natural product biosynthesis, biology, and pharmacology. However, despite the success with macromolecules, the labeling of natural products has not been adequately explored with fewer examples.

This perspective will discuss current strategies for labeling natural products and their applications in natural product research (Figure 1). We will limit the scope of this perspective to the labeling of polyketides (PKs) and non-ribosomal peptides (NRPs), which are the major classes of natural products noted for their functional and chemical diversity. We will also limit our discussion of bioorthogonal chemical transformations to the most widely used azide-alkyne [3+2] cycloaddition reaction, although many strategies and applications can also be applied to other bioorthogonal pairs (Prescher and Bertozzi, 2005; Sletten and Bertozzi, 2009).

## Labeling of PKs/NRPs through chemical synthesis

Chemical synthesis, such as total organic synthesis or semi-synthesis, is a well-adopted approach to install a “clickable” functionality on molecular scaffolds of natural products. The resulting orthogonally functionalized natural product analogs can be further modified through facile and selective chemical transformations for diversification, and more interestingly, for application in activity-based protein profiling experiments. For example, Cravatt and Sorensen's groups chemically synthesized a library of spiroepoxides inspired by natural products such as fumagillin, lumanicin D, and FR901464, and they performed *in situ* proteome reactivity profiling to determine the protein targets (Evans et al., 2005). Particularly, an alkyne handle was included in the scaffold of synthetic small molecule chemical probes, and two downstream azide-modified reporters were used: a rhodamine-azide reporter for initial protein target visualization by in-gel fluorescence scanning, and a trifunctional rhodamine-biotin-azide reporter for protein target enrichment, chromatographic purification, and further mass spectrometry analysis to determine the identity of the protein target. Similarly, Sieber's group synthesized alkyne-tagged β-lactams as selective chemical probes for the identification of bacterial enzymes involved in antibiotic resistance (Staub and Sieber, 2008, 2009). These artificial lactam probes were stable enough to resist β-lactamase hydrolysis, and were successfully used to detect and monitor the activities of several resistance associated proteins by fluorescence scanning and mass spectrometry analysis. It is notable that the sterically inconspicuous alkyne tag allowed the introduction of the bulky reporter group after enzyme binding and cell preparation, enabling the click chemistry-based analysis of proteins modified by tagged natural products in living cells. These results suggest that development of orthogonally functionalized natural products will help with studying the mode of action of natural products and aid in the discovery of new drug targets for customized therapeutic interventions. Alternatively, an azido functionality has also been introduced into the molecular scaffolds of natural products. For example, Sulikowski's group installed an azido handle into apoptolidins through chemical esterification of apoptolidins A and H obtained from microbial fermentation (DeGuire et al., 2015). These azido-functionalized analogs were shown to be as potent as their parent apoptolidins when evaluated by a cell viability assay. In addition, the cellular localization of these azido-labeled analogs in H292 human lung carcinoma cells were successfully visualized and identified using an alkyne-containing fluorescent reporter.

## Labeling of PKs/NRPs through biosynthesis

### Biosynthetic logic for PKs and NRPs

PKs, NRPs and their hybrids are major families of natural products with remarkable structural diversity and medicinal potential. These natural products are formed through the controlled assembly of simple biosynthetic building blocks with diverse tailoring reactions (Fischbach and Walsh, 2006; Hertweck, 2009). PK backbones are constructed by repeated condensations of acyl-CoAs catalyzed by polyketide synthases (PKSs) containing the core catalytic domains of ketosynthase (KS), acyltransferase (AT), and acyl carrier protein (ACP); and NRPs are assembled by condensations of amino acid monomers catalyzed by non-ribosomal peptide synthetases (NRPSs) containing the core catalytic domains of adenylation (A), condensation (C) and thiolation (T) (Fischbach and Walsh, 2006). A thorough understanding of PK/NRP biosynthetic machinery, particularly the substrate promiscuity, facilitates the installation of clickable functionalities onto diverse molecular scaffolds of PKs/NRPs through biosynthesis.

### Labeling of PKs/NRPs through precursor directed biosynthesis

Precursor directed biosynthesis (PDB) has been widely used to tag biomolecules, such as proteins, glycans, and nucleic acids, based on their promiscuous biosynthetic machinery (Grammel and Hang, 2013). Analogously, this approach represents a promising alternative to chemical synthesis for introducing a unique tag into natural product backbones with the incorporation of unnatural precursors. Depending on the relaxed substrate specificity of PKS and NRPS machinery, precursors with a bioorthogonal handle can be incorporated at the loading, extending, or tailoring stage of the biosynthetic pathways. Most downstream biosynthetic enzymes are expected to tolerate tagged biosynthetic intermediates, yielding predictable labeled natural products (Harvey and Khosla, 2012).

Based on the relaxed substrate specificity of the PKS at the loading stage, Khosla's group utilized the PDB approach to make an orthogonally functionalized erythromycin analog: 15-propargyl erythromycin A (Harvey et al., 2012). After they fed a synthetic terminal alkyne-tagged precursor that mimicked the natural diketide starter unit into the engineered biosynthetic pathway of erythromycin in *Escherichia coli*, the precursor was incorporated into the polyketide scaffold by the KS domain from module 2 of the 6-deoxyerythronolide B synthase (DEBS). This novel erythromycin analog showed comparable antibiotic potency as the clinically used erythromycin A in several bioassays.

The strategy of extender unit engineering, particularly based on a promiscuous AT domain that is native or achieved through site-directed mutagenesis, has also led to the introduction of a clickable functionality into PKs and PK-NRP hybrids. Although malonyl-CoA and methylmalonyl-CoA are the most common extender units for PKSs, atypical extender units have been found to be incorporated into PKs and PK-NRP hybrids by natively promiscuous AT domains. For example, it has been revealed that the AT domain embedded in AntD from the antimycin biosynthetic pathway tolerated a wide range of atypical fatty acyl extender units with different chain lengths and modifications (Sandy et al., 2012; Yan et al., 2012, 2013). Based on the relaxed substrate specificities of AntD-AT and AntE (a reductase/decarboxylase homolog to generate acylmalonyl-CoAs), Liu's group biosynthesized many terminal alkyne-tagged antimycin analogs after feeding alkynoic acids into the culture of *Streptomyces sp*. NRRL 2288 ΔantB, an engineered antimycin producer. Alternatively, an engineered AT domain with an altered substrate profile has also been used for incorporating a clickable functionality. Sanchez-Garcia and Schulz's groups found that the single V295A mutation of the AT domain from module 6 of DEBS enabled the incorporation of 2-propargylmalonyl instead of methylmalonyl as the extender unit, yielding a new orthogonally functionalized erythromycin analog: 2-propargylerythromycin in *Saccharopolyspora erythraea* (Sundermann et al., 2012). This AT engineering strategy could be possibly adopted for the engineering of other extending ATs, resulting in the site-selective introduction of a tagged extender unit into additional PKs or PK-NRP hybrids.

Additionally, clickable functionalities can also be installed onto the scaffolds of PKs/NRPs at the tailoring stage of the biosynthetic pathways. For example, a promiscuous tailoring AT, AntB from the antimycin biosynthetic pathway utilized terminal alkyne-containing precursors, resulting in terminal alkyne-labeled antimycins both *in vitro* and *in vivo* (Sandy et al., 2013; Yan et al., 2013). One of the alkyne-functionalized antimycin analogs was further incubated with Hela cells, followed by a reaction with an azide-containing fluorescent reporter for visualization. The efficient binding of this antimycin analog to Hela cells indicated that the installation of a terminal alkyne functionality had no significant effect on antimycin to recognize its protein target. In a different system, Jakeman's group fed *O*-propargyl-l-serine as the sole nitrogen source to a culture of *Streptomyces venezuelae* ISP5230, and the terminal alkyne functionality was installed onto the jadomycin scaffold non-enzymatically (Dupuis et al., 2012). An eight-membered library of jadomycin triazoles was further generated through subsequent reactions with a series of azides and the anti-cancer and antibacterial activities of these new compounds were evaluated. Using a similar approach to generate natural product derivatives for drug screening, Walsh's group synthesized carbohydrate-modified cyclic peptides utilizing a promiscuous thioesterase and a facile click chemistry-based chemical modification (Lin and Walsh, 2004). They first targeted the *in vitro* enzymatic synthesis of terminal alkyne-tagged cyclic tyrocidine derivatives through enzymatic macrocyclization of synthetic linear peptide *N*-acetyl cysteamine (SNAC) thioesters using the promiscuous thioesterase domain from the tyrocidine synthetase. The subsequent conjugation to 21 azido sugars via copper(I)-catalyzed cycloaddition yielded glycosylated cyclic peptides, some of which showed an improved therapeutic index compared to the natural tyrocidine. This chemoenzymatic approach offered several advantages over purely chemical or enzymatic synthesis. On the one hand, enzymes with relaxed substrate specificity could catalyze reactions such as macrocyclization which were difficult to achieve through chemical synthesis; on the other hand, chemical synthesis was more flexible than enzymatic synthesis. For example, enzymatic glycosylation suffered from the lack of promiscuous glycosyltransferases and corresponding glycosyl donor substrates. The chemoenzymatic strategy thus combines the strengths of both chemical and enzymatic approaches and can be widely used for modification of other natural products to search for new therapeutics.

### Labeling of natural products through *de novo* biosynthesis

**Figure 2:**
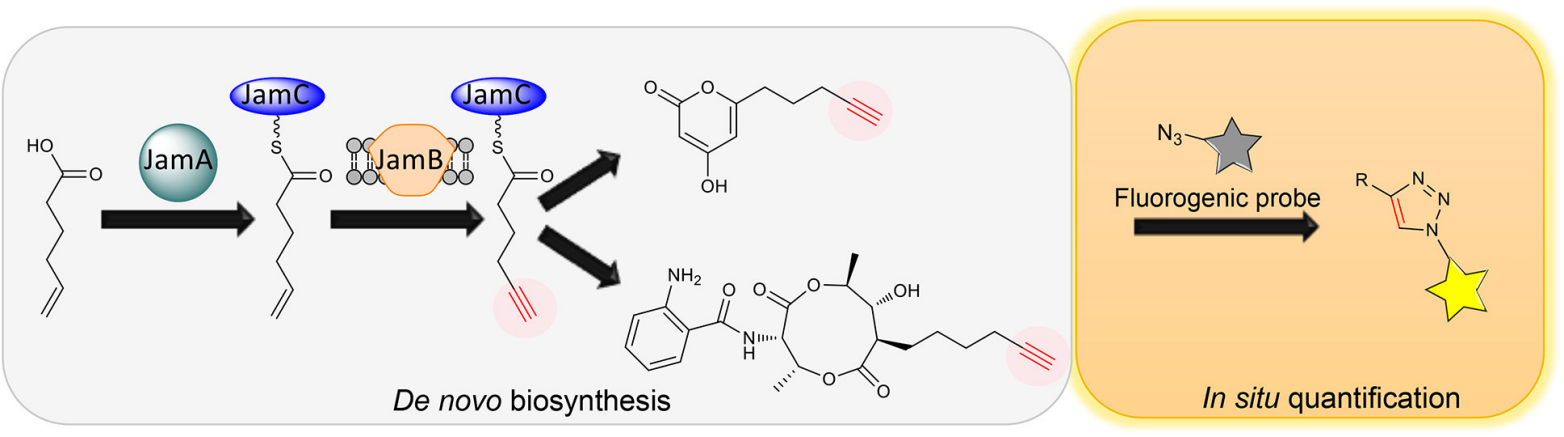
**Schematic of *de novo* biosynthesis of terminal alkyne-tagged natural products and its potential application for *in situ* quantification**.

The coexistence of diffusible precursors and final products with the same chemical handle introduces significant background in the PDB production system, making it incompatible with *in situ* bioorthogonal chemical transformations. To overcome the limitation of PDB for tagging natural products, our group recently developed a *de novo* biosynthesis platform to label natural products with a terminal alkyne functionality without the feeding of alkynoic precursors (Figure 2) (Zhu et al., 2015). Inspired by the discovery of gene clusters for jamaicamides and carmabins, which are terminal alkyne-bearing PK-NRP hybrids from marine cyanobacteria, we first characterized the terminal alkyne biosynthetic machinery consisting of JamABC, an acyl-ACP synthetase, a membrane-bound desaturase, and an ACP, respectively. In particular, we reconstituted the activities of JamABC by *in vitro* biochemical analysis and confirmed JamB to be the first terminal acetylenase that functions in a microbial PKS-NRPS pathway to install a terminal alkyne functionality. The biochemical characterization of JamABC resulted in a compelling sequence of chemical steps for generating the hexynoic starter unit for jamaicamides: 5-hexenoic acid is activated with ATP and loaded onto JamC by JamA, followed by the modification catalyzed by JamB before the priming of the PKS. A portable tri-gene cassette containing *jamABC* was thus identified that could be used to install the terminal alkyne functionality into various molecular scaffolds of other natural products through biosynthetic pathway engineering. As a proof of concept, we first targeted the biosynthesis of terminal alkyne-bearing PKs through starter unit engineering. A type III PKS HsPKS1 was chosen to assemble polyketide scaffolds due to its promiscuity in selecting starter units. The introduction of JamABC as well as HsPKS1 into *E. coli* successfully resulted in the generation and incorporation of the terminal alkyne into PKs, confirming the function of *jamABC* in the model organism *E. coli* and demonstrating the feasibility of *de novo* synthesizing terminal alkyne-labeled natural products by starter unit engineering. In addition, we explored extender unit engineering, an alternative strategy for incorporating an alkynoic extender unit into the molecular scaffolds of PKs. Taking the advantage of the promiscuous AntD-AT and AntE from the antimycin biosynthetic machinery mentioned above, we *de novo* biosynthesized antimycin analogs tagged with a terminal alkyne functionality using an engineered *E. coli* strain containing *jamABC* and the minimal set of genes necessary for antimycin dilactone scaffold assembly.

## Is there a general way to synthesize tagged PKs/NRPs?

It is often challenging to obtain tagged PKs/NRPs through total organic synthesis because of their structural complexity and stereochemical centers, or through semi-synthesis because of their chemical lability and the limited supply. Even though chemists could design feasible routes to synthesize a tagged natural product, these routes are not likely to be generalizable to other natural products because of the diversity found in the functional group arrays and scaffold architectures of PKs/NRPs. Alternatively, biosynthesis is a promising approach to tag the majority of natural products, particularly PKs and NRPs, due to the modularity and co-linearity of their biosynthetic machinery. A tagged building monomer can be incorporated into the molecular scaffold of targeted natural products using the native or engineered promiscuous biosynthetic machinery of natural products. Various pathway-engineering strategies can be generalized to synthesize tagged PKs/NRPs based on the features of the natural products of interest and the clickable precursors. For example, an alkynoic precursor can be introduced to label many natural product families, including but not limited to PKs, PK-NRP hybrids, lipopeptides, and lipoglycopeptides, through strategies such as starter unit, extender unit, and tailoring unit engineering. Particularly, starter unit engineering can be generalized to label PKs and lipopeptides that naturally contain fatty acyl starter units. Extender unit engineering requires a promiscuous AT domain that is readily achievable through domain swapping or site-directed mutagenesis, and can possibly lead to the regiospecific introduction of an alkyne functionality into any PKs and PK-NRP hybrids inside living cells. In addition, since acylation/lipidation is one of the most common modification reactions in natural product biosynthesis, and the responsible transferases typically have relaxed substrate specificities toward various acyl donors, it is highly likely that a functionalized acyl chain can be incorporated into natural product scaffolds in a general fashion through promiscuous tailoring acyltransferases.

## Does natural product labeling have a broader impact beyond current known applications?

The limited examples of natural product labeling have been focused on structural diversification of natural products for drug screening and mode of action studies through identification of protein targets and cellular localization. Indeed, broader applications of natural product labeling can be foreseen, ranging from natural product discovery to biology and enzymology. Firstly, a general approach to tag PKs, NRPs and PK-NRP hybrids with chemical handles would greatly accelerate the purification process: the tagged natural products can be enriched and isolated using functionalized resins via bioorthogonal reactions. Secondly, the tagged natural products can be easily visualized and traced, providing a unique opportunity to assess the natural roles of natural products. This is an emerging research field as it is now being realized that many natural products are molecules of adaptation that are produced for specific physiological or social reasons (O'Brien and Wright, 2011). Despite a long history of natural product research, the natural roles of these compounds are only just now beginning to be understood. Last but not the least, the *de novo* biosynthesis of tagged natural products would permit *in situ* detection and quantification of natural products in producing cell cultures through coupling with fluorogenic probes (Shieh et al., 2012, 2014) (Figure 2). This strategy would lead to a natural product-based, quantitative, and high-throughput screening method, which can be leveraged to understand and engineer the biosynthesis of natural products for overproduction and diversification.

### Conflict of interest statement

The authors declare that the research was conducted in the absence of any commercial or financial relationships that could be construed as a potential conflict of interest.
